# Using the COM-B model to identify barriers to and facilitators of evidence-based nurse urine-culture practices

**DOI:** 10.1017/ash.2023.142

**Published:** 2023-03-31

**Authors:** Sonali D. Advani, Ali Winters, Nicholas A. Turner, Becky A. Smith, Jessica Seidelman, Kenneth Schmader, Deverick J. Anderson, Staci S. Reynolds

**Affiliations:** 1 Division of Infectious diseases, Department of Medicine, Duke University School of Medicine, Durham, North Carolina; 2 Duke Center for Antimicrobial Stewardship and Infection Prevention, Durham, North Carolina; 3 Duke University School of Nursing, Durham, North Carolina; 4 Division of Geriatrics, Duke University School of Medicine, Durham, North Carolina; 5 Geriatric Research and Education Clinical Center, Durham Veterans Administration Medical Center, Durham, North Carolina

## Abstract

Our surveys of nurses modeled after the Capability, Opportunity, and Motivation Model of Behavior (COM-B model) revealed that opportunity and motivation factors heavily influence urine-culture practices (behavior), in addition to knowledge (capability). Understanding these barriers is a critical step towards implementing targeted interventions to improving urine-culture practices.

Nurses play an important role in influencing culture practices and antimicrobial prescribing, but they are often overlooked in stewardship interventions.^1^ How nurses communicate a patient’s condition can impact whether the clinician orders a urine culture and/or prescribes antibiotics.^2^ However, prior surveys of nurses have revealed that knowledge related to evidence-based indications for ordering urine cultures may be low.^3,4^ In addition, poor collection techniques may lead to contaminated or false-positive results, further complicating the clinician’s ability to interpret a urine-culture result.^5^

Prior data related to nurse-driven urine-culture practices have primarily focused on assessing knowledge, with little investigation into social, environmental, and cultural barriers that influence these practices.^4,6^ The Capability, Opportunity, Motivation and Behavior (COM-B) model examines the interactions among 3 components: capability, opportunity, and motivation on behavior. In this study, we applied the COM-B model^7^ to understand barriers to and facilitators of evidence-based urine-culture practices (behavior) by nurses in inpatient settings.

## Methods

### Design

We conducted cross-sectional surveys of nurses between August 1 and October 5, 2022. This study was deemed a quality improvement project by the Duke University Institutional Review Board.

### Setting

These surveys were conducted in 3 inpatient units (37-bed neuroscience intensive care unit, 37-bed neuroscience stepdown unit, and a 32-bed urology–gynecology oncology unit) at Duke University Hospital, a 1,048-bed academic medical center in Durham, North Carolina.

### Survey instrument and distribution

We adapted a previously validated survey instrument using the COM-B model.^3,8^ This survey included questions on the role of the nurse (8 questions), capability or knowledge (16 questions and sub-questions), opportunity (4 questions) and motivation factors (5 questions) related to urine-culture practices, and 1 additional question (Supplementary Material 1: Survey). Of the 16 knowledge questions, 12 were related to indications and 4 were related to collection techniques. The accuracy of the responses was assessed based on the 2009 Infectious Diseases Society of America (IDSA) catheter-associated urinary tract infection (CAUTI) guidelines and the 2019 asymptomatic bacteriuria guidelines.^9,10^ Correct answers received a score of 1 point, and all correct answers were added for a total maximum score of 16 for knowledge (capability). Infection prevention staff electronically distributed the surveys via Qualtrics to nurses using Quick Response codes. Participation was voluntary and anonymous.

### Data analysis

We reported means for continuous variables and percentages for categorical variables. We compared the difference in mean total knowledge scores across different groups using the Student *t* test and analysis of variance, as appropriate. We measured opportunity and motivation on a 4-point Likert scale from 1 (strongly disagree) to 4 (strongly agree). We also tested for correlation between capability and motivation using correlation plots and the Kruskal γ for correlation.

## Results

We received 114 responses to our survey, with a response rate of 45.5%. Mean respondent age was 30.7 years (SD, 9.9), 88.6% identified as women, with 6.24 mean years of experience. Most nurses held a bachelor’s of science in nursing degree (n = 98, 86%) and worked on day shift (n = 61, 53.5%).

### Capability

**Fig. 1.**
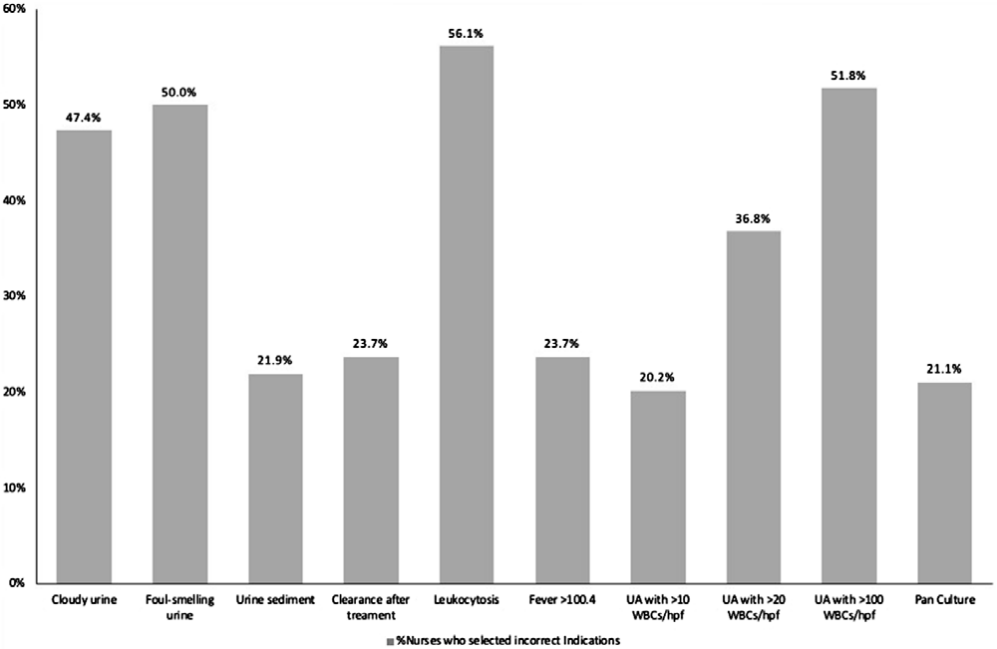
Comparison of nursing knowledge (capability) scores for evidence-based urine-culture indications and collection techniques by question type.

The mean total knowledge score for indications and collection techniques was 9.93 (SD, 2.9) of 16. The mean knowledge score for indications only was 7.02 (SD, 2.57) of 12. There were no differences in mean total knowledge scores between units based on gender or type of shift. However, nurses with a master’s degree (13.75; *P* = .027) and >20 years of experience scored higher (11.27; *P* = .02) than other nurses (Supplementary Material 2). Comparison of knowledge scores by question type is shown in Figure 1.

### Opportunity

**Fig. 2:**
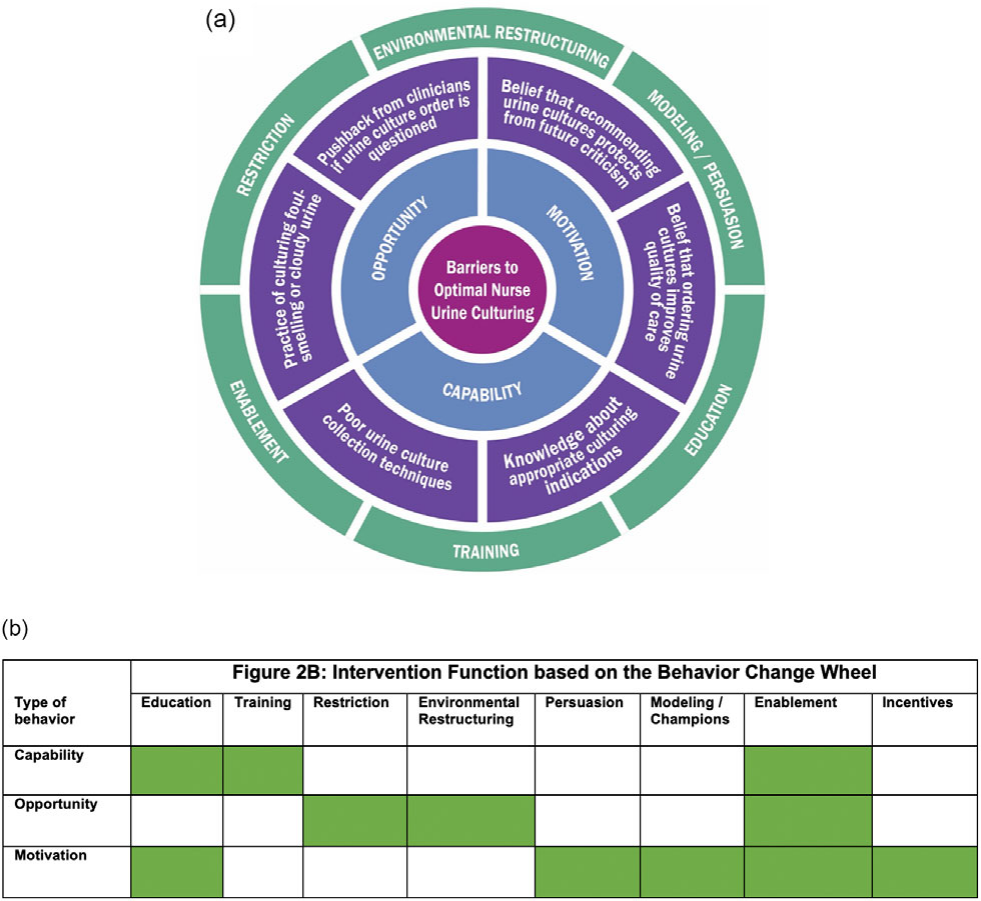
(A) Barriers to evidence-based urine culture faced by nurses using the Capability Opportunity Motivation–Behavior (COM-B model). (B) Intervention functions based on the behavior change wheel.

On a 4-point Likert scale, nurses reported that they were likely to receive pushback from clinicians when they questioned a urine-culture order (mean, 2.23; SD, 0.67) (Fig. 2A). Nurses were also likely to request a urine-culture order if a patient’s urine was cloudy or foul smelling (mean, 2.73; SD, 0.75) (Fig. 2A). In terms of facilitators, most nurses reported that they provided education to patients on the appropriate way to collect a clean-catch urine specimen (mean, 3.48; SD, 0.50) and had the resources necessary to make an informed recommendation to clinicians regarding appropriate urine-culture orders (mean, 3.12; SD, 0.53).

### Motivation

Most nurses felt that ordering urine cultures improved the quality of care (mean, 2.75; SD, 0.79) and that asking a clinician for a urine-culture order protected them against future criticism (mean, 2.12; SD, 0.77) (Fig. 2A). In terms of facilitators, nurses felt confident in asking for clarification about a urine-culture order (mean, 3.14; SD, 0.58) and ordering a urine culture if they felt that the test was warranted (mean, 3.22; SD, 0.53).

### Correlation

Confidence in asking for clarification about a urine-culture order was not related to capability (γ correlation, 0.10; 95% CI, −0.15 to +0.35). Similarly, confidence related to requesting a urine culture was not related to capability (γ correlation, 0.02; 95% CI, −0.23 to +0.27).

## Discussion

Our findings highlight specific barriers to evidence-based urine-culture practices faced by nurses in a large academic medical center. Our data suggest that knowledge (ie, capability) alone, is insufficient to assure adherence to recommended urine-culture practices.^3,4^ We further emphasize that the other components of the COM-B model, namely opportunity (O) and motivation (M) factors, have a significant impact on nurse urine-culture behavior (B) in inpatient settings.

In the context of the COM-B model, capability refers to having the knowledge to engage in the desired behavior: evidence-based urine culturing. In a previous survey of nurses in a similar sized, large, academic medical center, knowledge score for appropriate indications was 6.5 (vs 7.02 in our study).^3^ Specifically, fewer nurses selected incorrect indications: foul-smelling urine (50 vs 72%) and cloudy urine (47.4% vs 60%) on our surveys compared to our prior study.^3^ However, despite this higher capability or knowledge, nurses reported that it was an ingrained practice to order a urine culture for cloudy or foul-smelling urine, likely to due to external factors or peer pressure (opportunity).

Another factor that heavily influences behavior is beliefs about consequences and overall confidence around the intended behavior (motivation). Specifically, motivators like “sending urine cultures helps improve the quality of care provided to patients” and “asking a physician to order a urine culture helps protect me from future criticism” influence urine-culture practices in inpatient settings. This study is the first to highlight opportunity and motivation as factors that heavily influence nurse urine-culture behavior. Additionally, motivation or confidence did not correlate with knowledge scores; this finding underscores that interventions that solely focus on improving knowledge do not influence motivation. Opportunity and motivation barriers require specific interventions such as environmental restructuring, enablement, and modeling (champions), which can be identified using the “behavior change wheel” (Fig. 2B).^7^

Our study had several limitations. Our response rate was 45.5%, which is consistent with similar studies using electronic surveys.^3^ The nurses we surveyed may differ from nurses working in other settings (eg, general medicine, geriatrics, transplant units, etc). Surveys were performed by infection prevention staff, which may have biased results. Additionally, generalizability is limited because this study was performed in 3 units of a large, academic medical center.

In conclusion, focusing on knowledge alone is insufficient to improve evidence-based urine-culture (behavior) practices. Opportunity and motivation play key roles in influencing urine-culture (behavior) practices. Healthcare systems should include nurses in stewardship efforts and should consider interventions that target opportunity and motivation barriers to improve urine-culture practices.

## Supplementary material

For supplementary material accompanying this paper visit http://doi.org/10.1017/ash.2023.142. click here to view supplementary material
