# Supplementary material for: Using the COM-B model to identify barriers to and facilitators of evidence-based nurse urine-culture practices
Source: Antimicrob Steward Healthc Epidemiol. 2023 Mar 31;3(1):e62. doi: 10.1017/ash.2023.142 (PMC10073011; doi:10.1017/ash.2023.142)
Supplement: Supplementary file 1 [file ashsup.zip › S2732494X23001420sup001.docx]

**Supplement.** Nursing survey of urine culture practices

***Demographic Questions***

1. Please think of an 8-digit unique# (such as someone’s birthday); this # will be used to match your pre- and post-survey responses: ______________________

2. What unit do you work on? 7DCT 8DCT 6300

3. What is your age in years? _____________________

4. How many years of nursing experience do you have? ___________

5. What shift do you **primarily** work? Day Night Rotating

6. Please select your role: CNI CNII CNIII CNIV CTL NM

7. Please select your highest level of nursing education: ADN BSN MSN DNP/PhD Other

8. To which gender identify to you most identify? Woman Man Transgender Gender Variant Not listed: _____ Prefer to not answer

***Knowledge/Capability Questions (adapted from Advani et al., 2019)***

1. For which of the following indications would you send a urine culture in a **CATHETERIZED** patient? Check all that apply.

- Cloudy urine
- Foul-smelling urine
- Urine sediment in the foley tubing or bag
- New confusion in an elderly patient
- Peri-urologic surgery with anticipated mucosal bleeding such as TURP
- Follow-up to check for clearance of urinary tract infection
- Leukocytosis with WBC>10,000
- Single temperature to 100.4 in an immunocompetent patient
- Pyuria with >10 WBCs/hpf on urinalysis
- Pyuria with >20 WBCs/hpf on urinalysis
- Pyuria with >100 WBCs/hpf on urinalysis
- Other (enter comment)

1. You are caring for a 50 year old female with recent GI perforation s/p exploratory-laparotomy, who presents with a new fever of 101.4^o^F on post-op day 2. She has a urinary catheter but no signs of obstruction or flank pain. Which of the following actions would you take?
2. Recommend the clinician order pan-cultures, including blood, urine, and sputum culture; chest x-ray; and abdominal imaging.
3. Recommend the clinician first obtain abdominal imaging and blood cultures given recent surgery, followed by chest x-ray and sputum cultures if respiratory symptoms, followed by urine cultures if other tests were negative

3. Your neutropenic patient has had a urinary catheter in for 4 days. They now have a fever (101.8^o^F) and suprapubic pain. The clinician orders a urinalysis and urine culture from the Foley. How should the urine culture be collected from the Foley? Choose the best response.

- 1. Scrub the aspiration port for 15 seconds and allow to air dry. Attach the vacutainer to the port and aspirate urine into the grey and yellow tubes.
  2. On the drainage bag, scrub the drainage spigot for 15 seconds and allow to air dry. Drain the urine into a sterile culture cup and send to lab.
  3. Scrub the aspiration port for 5 second and attach the vacutainer. Aspirate urine into the grey tube.
  4. Urine cultures should never be drawn from a Foley catheter.

4. Your patient has a burning sensation with urination and flank pain. The clinician orders a clean catch urinalysis and urine culture. It is appropriate to collect the urine from a bedpan or hat as long as it has never been used before.

1. True
2. False

5. Which of the following are appropriate ways to collect urine specimens? Choose all that apply.

1. From the urinary catheter port, only after it has been scrubbed for at least 15 seconds and allowed to dry.
2. A clean catch taken from a bedpan or hat.
3. From the urinary catheter drainage bag as long as the spigot has been scrubbed for 15 seconds.
4. An intermittent catheterization.
5. From the suction canister if the patient has an external catheter.

6. After you have collected a urine specimen, when should it be transported to the lab?

1. By the end of your shift
2. Within 2 hours of obtaining the specimen
3. As soon as possible after obtaining the specimen
4. Within 48 hours as long as it has been refrigerated

***Opportunity Questions***

Please respond to the below statements using the provided Likert-scale.

1. I have the resources necessary to make an informed recommendation to clinicians regarding appropriate urine culture ordering.
   1. 1=Strongly Disagree
   2. 2=Disagree
   3. 3=Agree
   4. 4=Strongly Agree
2. I have received pushback from clinicians when I have questioned their urine culture ordering practices.
   1. 1=Strongly Disagree
   2. 2=Disagree
   3. 3=Agree
   4. 4=Strongly Agree
3. If my patient’s urine is cloudy or foul smelling, it is our practice to ask the clinician for a urine culture order.
   1. 1=Strongly Disagree
   2. 2=Disagree
   3. 3=Agree
   4. 4=Strongly Agree
4. When a clinician orders a clean catch urine sample from my patient, I am able to provide education to my patient on the appropriate way to collect the urine.
   1. 1=Never
   2. 2=Sometimes
   3. 3=Most of the time
   4. 4=Always

***Motivation Questions***

1. Patients with a urinary catheter should have frequent urine cultures drawn since they are at a higher risk of infection.
   1. 1=Strongly Disagree
   2. 2=Disagree
   3. 3=Agree
   4. 4=Strongly Agree
2. Sending urine cultures helps improve the quality of care provided to patients.
   1. 1=Strongly Disagree
   2. 2=Disagree
   3. 3=Agree
   4. 4=Strongly Agree
3. Asking a clinician to order a urine culture helps protect me from future criticism.
   1. 1=Strongly Disagree
   2. 2=Disagree
   3. 3=Agree
   4. 4=Strongly Agree
4. When I feel that a urine culture is not indicated, I feel confident in asking for clarification from the clinician.
   1. 1=Not confident at all
   2. 2=Slightly confident
   3. 3=Fairly confident
   4. 4=Extremely confident
5. When I feel that my patient should have a urine culture sent, I feel confident requesting this order from the clinician.
   1. 1=Not confident at all
   2. 2=Slightly confident
   3. 3=Fairly confident
   4. 4=Extremely confident

**Additional Question:**

What questions or practices do you need clarity on regarding urine cultures?
