# Supplementary material for: Using the COM-B model to identify barriers to and facilitators of evidence-based nurse urine-culture practices
Source: Antimicrob Steward Healthc Epidemiol. 2023 Mar 31;3(1):e62. doi: 10.1017/ash.2023.142 (PMC10073011; doi:10.1017/ash.2023.142)
Supplement: Supplementary file 1 [file ashsup.zip › S2732494X23001420sup002.docx]

**Supplement 2. Overview of survey responses**

| **Knowledge Questions** | **Mean (SD)** | **p-value** |
| --- | --- | --- |
| Total Knowledge score (Indications + Collection) | 9.93 (2.9)  Range 2-16 |  |
| Knowledge score (Indications only) | 7.02 (2.57) |  |
| Differences between shifts |  | .099 |
| Day shift | 10.21 (2.8) |  |
| Night shift | 10.58 (3.0) |  |
| Rotating | 9.06 (2.8) |  |
| Differences between units |  | .299 |
| Neuroscience intensive care unit (n=57) | 9.6 (2.9) |  |
| Neuroscience stepdown (n=29) | 10.62 (3.1) |  |
| Urology-gynecology oncology unit (n=27) | 9.81 (2.8) |  |
| Differences between degrees |  | .027 |
| Diploma (=2) | 12.5 (4.9) |  |
| ADN (n=10) | 9.6 (2.8) |  |
| BSN (n=98) | 9.76 (2.8) |  |
| MSN (n=4) | 13.75 (1.5) |  |
| Differences between gender |  | .244 |
| Men (n=10) | 11.2 (2.8) |  |
| Women(n=101) | 9.82 (2.9) |  |
| Gender variant (n=1) | 6 |  |
| Prefer to not answer (n=2) | 11.0 (2.8) |  |
| Differences between age ranges |  | .003 |
| 20-29 (n=72) | 9.14 (2.8) |  |
| 30-39 (n=26) | 11.54 (2.3) |  |
| 40-49 (n=5) | 11.2 (2.6) |  |
| 50-59 (n=10) | 10.6 (3.6) |  |
| 60-69 (n=1) | 12 |  |
| Differences between years of experience |  | .002 |
| 2 years or less (n=53) | 8.75 (2.5) |  |
| 3-5 years (n=24) | 10.33 (2.8) |  |
| 6-8 years (n=12) | 10.83 (3.6) |  |
| 9-10 years (n=5) | 12.2 (1.1) |  |
| 11-15 years (n=8) | 11.88 (1.8) |  |
| 16-20 years (n=1) | 10 |  |
| Greater than 20 years (n=11) | 11.27 (3.3) |  |
|  |  |  |
